# Congenital Syphilis: A Re-Emerging but Preventable Infection

**DOI:** 10.3390/pathogens13060481

**Published:** 2024-06-06

**Authors:** Serena Salomè, Maria Donata Cambriglia, Giovanna Montesano, Letizia Capasso, Francesco Raimondi

**Affiliations:** Division of Neonatology, Department of Translational Medical Sciences, University of Naples Federico II, Via Pansini 5, 80131 Naples, Italy; d.cambriglia@gmail.com (M.D.C.); giovanna.montesano@hotmail.com (G.M.); letizia.capasso@unina.it (L.C.); raimondi@unina.it (F.R.)

**Keywords:** congenital syphilis, syphilis, prevention, maternal screening

## Abstract

Congenital syphilis presents a significant global burden, contributing to fetal loss, stillbirth, neonatal mortality, and congenital infection. Despite the target established in 2007 by the World Health Organization (WHO) of fewer than 50 cases per 100,000 live births, the global incidence is on the rise, particularly in low- and middle-income regions. Recent data indicate a rate of 473 cases per 100,000 live births, resulting in 661,000 total cases of congenital syphilis, including 355,000 adverse birth outcomes such as early fetal deaths, stillbirths, neonatal deaths, preterm or low-birth-weight births, and infants with clinical congenital syphilis. Alarmingly, only 6% of these adverse outcomes occurred in mothers who were enrolled, screened, and treated. Unlike many neonatal infections, congenital syphilis is preventable through effective antenatal screening and treatment of infected pregnant women. However, despite available screening tools, affordable treatment options, and the integration of prevention programs into antenatal care in various countries, congenital syphilis remains a pressing public health concern worldwide. This review aims to summarize the current epidemiology, transmission, and treatment of syphilis in pregnancy, as well as to explore global efforts to reduce vertical transmission and address the reasons for falling short of the WHO elimination target.

## 1. Introduction

Syphilis, caused by the bacterium *Treponema pallidum* subspecies *pallidum* (*T. pallidum*), is a sexually transmitted infection (STI) that can be acquired horizontally through contact with infected skin lesions (chancre, mucus patch, or condyloma latum) or vertically from mother to fetus, primarily through transplacental transmission during pregnancy or, less commonly, during delivery through contact with a maternal lesion. Infection can also occur after birth, for example, during breastfeeding from lesions of the maternal nipple [1]. Therefore, syphilis in newborns can be defined as *congenital* or *prenatal* if contracted by the fetus transplacentally or *connatal* if contracted by the newborn through the birth canal.

Since the advent of penicillin, syphilis has become an infectious disease that is not only preventable but also treatable, leading to a decline in Western countries like the U.S. after 1990, with a nadir in 2001. Despite this, though once thought to be eradicated, there has been a resurgence of this pathogen worldwide [2]. First described in 1497 by Gaspar Torella [3], congenital syphilis (CS) remains a significant cause of stillbirths globally, with serious adverse effects occurring in up to 80% of cases [4] and associated morbidity and mortality among affected newborns [5]. Nevertheless, simple, cost-effective antenatal screening and treatment of infected pregnant women could prevent and ideally eliminate CS. Despite the availability of screening tools, affordable treatment options, and the integration of prevention programs into antenatal care in many countries, CS persists as a public health challenge, with increasing rates observed in many regions [6,7]. Timely pre- and perinatal screening, followed by the appropriate treatment of maternal syphilis infection with penicillin, is crucial to preventing the development of CS [8].

## 2. Epidemiology

Following the introduction of penicillin, there was a significant reduction in the incidence of syphilis in the U.S., with a 75% decrease by 1954 compared to 1944, followed by a further 90% reduction in 1975, accompanied by a corresponding decrease in CS rates from 368 cases per 100,000 to 37 cases per 100,000 [9]. By the late 1990s, global syphilis incidence was declining, reaching a nadir of 2.1 cases per 100,000 persons in 2001 [10]. This decline was primarily attributed to the widespread use of penicillin, which provided an effective and relatively inexpensive treatment, as well as effective prevention measures, such as increased condom use prompted by the AIDS epidemic.

However, over the past decade, global syphilis rates have surged, increasing by approximately 60% from 1990 to 2020. Syphilis, like many other STIs, disproportionately affects populations with limited access to healthcare, resulting in geographical, racial, and ethnic disparities [11,12]. While syphilis transmission has historically been more prevalent in at-risk populations marked by factors such as substance abuse, poor socioeconomic status, and disadvantage, it is increasingly affecting low-risk populations globally [13]. The lastest available European data are from 2022 and reported 35,391 confirmed cases, giving a crude notification rate of 8.5 cases per 100,000 population with 2540 of them reported in Italy (that is a rate of 4.3 per 100,000 population) [14].

Currently, syphilis remains a global health concern, with an estimated 7.1 million adults infected annually and an overall increase in syphilis among women of reproductive age. This is particularly significant as women play a crucial role due to negative sequalae and adverse maternal outcomes including CS. Approximately seven in every 1000 pregnant women worldwide are diagnosed with syphilis annually, with the highest burden observed in sub-Saharan Africa. These maternal syphilis cases led to a global incidence of 473 cases of CS per 100,000 live births, totaling 661,000 cases, including 355,000 adverse birth outcomes, such as 143,000 early fetal deaths and stillbirths, 61,000 neonatal deaths, 41,000 preterm or low-birth-weight births, and 109,000 infants with clinical CS [15]. Alarmingly, only 6% (23,000 cases) of these adverse birth outcomes occurred in mothers who were enrolled, screened, and treated.

Case rates are rising all over the world, especially in low- and middle-income settings. In fact, despite a marginal decline in the African region between 2007 and 2020, African countries had the highest rate of CS globally, accounting for 60% of cases. This can be attributed to high maternal prevalence and limited access to antenatal care. However, this trend is not exclusive to low-to-middle income countries (LMICs) as high-income countries (HICs) have also experienced a resurgence of CS over the past decade [7].

For instance, in the U.S., there has been a staggering 755% increase from 2012 to 2021, with a total of 3761 infected babies in 2022 corresponding to a national rate of 77.9 cases per 100,000 live births. Moreover, in 2022, there were 231 stillbirths and 51 infant deaths [16]. This increase underscores the failure of timely testing and adequate treatment, with substantial proportions of cases occurring across all geographic areas and racial and ethnic groups. Vulnerable populations, including minorities and disadvantaged groups, such as Native populations, African Americans, or Hispanic individuals, are particularly affected [16]. Similarly, in Europe, high prevalence is mainly reported in vulnerable populations with a higher proportion of persons using drugs and transactional sex along with insufficient coverage by preventative services [7]. According to the European Centre for Disease Prevention and Control (ECDC) [17], after a peak in 2019 with 73 cases (equal to an overall rate of 1.9 cases per 100,000 live births), there has been a recent decrease in CS cases even though underreporting is suspected. The lastest available data, from 2021, reported 47 cases in 23 E.U. countries, equal to an overall rate of 1.8 cases per 100,000 live births and ranging from 0.3 in Poland (1 case) to 22.2 in Bulgaria (13 cases). In HICs like the U.S., an increase in cases of CS diagnosed in late infancy and early childhood among international adoptees or refugees has also been recently reported [18].

Socioeconomic factors, cultural differences, and territorial disparities significantly influence the incidence of CS between countries, despite similar control strategies [19,20]. Additionally, the recent escalation in CS rates can be partially attributed to the redirection of public health resources to respond to the COVID-19 pandemic [21]. All these risk factors are summarized in Table 1.

Despite available epidemiological data indicating a global increase in CS cases, the true burden is likely underestimated due to poor surveillance systems, inadequate information systems, and high rates of unrecognized or asymptomatic infections [23]. Moreover, Mosley P et al. [24] underlined that variations in case definitions pose challenges for the meaningful comparison of surveillance data across countries (which have local and national surveillance mechanisms) and regions (as defined by the WHO). More robust surveillance and information systems are needed to accurately measure the current incidence of adverse birth outcomes due to syphilis.

Furthermore, the burden of CS remains underappreciated compared to other neonatal infections, despite being more frequent than human immunodeficiency virus (HIV) infection and tetanus [25]. Eliminating mother-to-child transmission (MTCT) of syphilis remains a global commitment, and in 2007, the World Health Organization (WHO) promoted a specific initiative setting a target of fewer than 50 cases per 100,000 live births by 2009 [26]. This initiative recommended political commitment and advocacy, increasing access to and quality of maternal and newborn health services, the screening of all pregnant persons and treatment of all positive cases and their partners, and establishing an underlying foundation of surveillance, monitoring, and evaluation. The programmatic targets to reach the goal were as follows: antenatal care coverage of at least 95%, a syphilis testing rate for pregnant women of at least 95% among those attending at least one antenatal care visit, and adequate syphilis treatment for at least 95% of pregnant women with syphilis seropositivity. However, neither of these targets have been met, highlighting the ongoing challenges in achieving effective prevention and control measures. In a recent WHO global progress report, CS is still the second leading cause of preventable stillbirth globally and a leading infectious cause of long-term disability globally; therefore, the WHO is still working towards eliminating MTCT of syphilis, together with HIV and hepatitis B [2].

Untreated syphilis has profound implications for maternal and neonatal health outcomes, with MTCT estimated to incur significant disability-adjusted life years and resulting in a cost of USD 3.6 million and global medical costs of USD 309 million (data derived from a Chinese study) [27].

## 3. Management during Pregnancy

Appropriate management during pregnancy is essential to prevent CS and improve maternal and neonatal health outcomes. Timely testing and treatment during pregnancy might have prevented almost nine of every 10 (88%) cases in 2022, and two of every five (40%) women who had a baby with CS did not receive prenatal care [16].

### 3.1. Clinical Manifestations

Syphilis in pregnant women occurs with the same clinical features of those detectable in nonpregnant individuals [28] and it is traditionally classified in four stages, that develop consecutively over time in untreated patients, and guide treatment and follow-up care [29].

The *primary* stage of syphilis may occur as a solitary chancre, indurated and ulcerative, with a clean base, which typically appears at the site of contact with the sex partner’s infectious lesion within 3 weeks after exposure and heal within 1 or 2 months. The chancre is usually painless and may occur at extragenital sites such as the perirectal area, the rectum itself, or the oral cavity. Multiple painful anogenital ulcers may also occur.

Clinical manifestations of *secondary* syphilis include a mild, nonpruritic rash, particularly on the palms and soles; fever; lymphadenopathy; mucosal lesions (e.g., mucous patches or condyloma latum); alopecia; periostitis; and occasionally hepatitis (often with high alkaline phosphatase values but minimally elevated aminotransferase levels) or nephritis. All these manifestations usually develop within 3 months of infection and can overlap with the primary stage in some cases, particularly in persons with human immunodeficiency virus (HIV) infection. Moreover, they have a broad differential diagnosis. Primary syphilis and secondary syphilis are the sexually transmissible stages of infection.

Resolution of the signs and symptoms of syphilis defines *latent* infection, which is detectable only by serologic testing in approximately 70% of untreated persons. Latent infection acquired within the preceding year is referred to as *early latent* syphilis, otherwise it is defined as *late latent* syphilis [30]. According to the WHO, the cut-off for differentiating between early and late latent syphilis is 2 years rather than 1 [31]. This is one of the fields where homogenous definitions are lacking. Syphilis may also be contagious in the early latent phase, as that is an asymptomatic stage occurring between the primary and secondary stages, but also after the resolution of secondary-stage lesions. In up to 24% of patients, early latent syphilis is interrupted by relapse with recurrent, infectious secondary lesions. The high proportion of cases of early latent syphilis suggests that primary syphilis and secondary syphilis frequently go unnoticed or are misidentified.

Asymptomatic or symptomatic neurologic involvement may occur during any stage of syphilis. CNS invasion by treponemes is accompanied by abnormal cerebrospinal fluid (CSF) findings in up to 50% of persons after early infection, even in the absence of clinical features (termed asymptomatic neurosyphilis). These CSF abnormalities typically resolve after the recommended therapy for early syphilis. Early clinical findings (i.e., early neurosyphilis or “neurorecurrence”) include meningitis, often a basilar form resulting in cranial-nerve abnormalities.

The *tertiary* stage with severe complications can develop several years to several decades after the initial infection in 30% of untreated cases, characterized by granulomatous lesions that affect multiple body organs with irreversible damage and can take the form of neurosyphilis (in which the brain or spinal cord is affected), cardiovascular syphilis (involving the aorta and heart), or late benign syphilis (primarily involving the skin). With widespread use of antibiotics, the occurrence of tertiary syphilis is less common now [32].

### 3.2. Maternal-to-Fetal Transmission

Maternal-to-fetal transmission (MTFT) of syphilis typically occurs during pregnancy through transplacental passage of *T. pallidum*. This transmission can happen at any trimester, predominantly during maternal infections that have disseminated. Infrequently, neonatal infection may result from exposure to syphilitic genital lesions during delivery [33]. While there are limited data on the exact sequence of fetal infection, it is believed to start with placental infection, followed by amniotic fluid infection, leading ultimately to hematological dysfunction, which may manifest as nonimmune hydrops. However, the precise sequence remains uncertain [34].

The likelihood of transmission is directly correlated with both the stage of maternal syphilis during pregnancy and the timing of infection acquisition during pregnancy. In untreated mothers with primary or secondary syphilis during the third trimester, the MTFT rate ranges from 60 to 100%, while in the latent stages it varies from 40% in early latent syphilis to less than 8% in late latent syphilis [28]. However, estimating the risk may be complicated by infections that predate pregnancy [35].

The concentration of *T. pallidum* in the blood peaks during the first two years after infection and gradually declines thereafter due to acquired immunity. Consequently, the risk of infecting sexual partners is highest during the first two years and diminishes afterward. However, the risk of maternal–fetal transmission persists beyond this period [28].

Although reporting practices for the adverse outcomes of untreated syphilis during pregnancy vary, the WHO suggests that any adverse outcome occurs in 75% of cases [2]. These adverse outcomes may include stillbirth and miscarriage in approximately 20% of cases and perinatal death in 15% of cases. Moreover, 20% of infants are congenitally infected, and in a further 20%, newborns presented with a low birth weight or were born premature and needed neonatal intensive care unit (NICU) admission [36].

Transmission of *T. pallidum* through breast milk is not documented. However, there is a possibility of syphilis transmission through direct contact with sores or skin lesions involving the nipple, areola, or breast tissue during breastfeeding or breast milk expression. Thus, breastfeeding is not contraindicated in perinatal syphilis, provided there are no lesions on the breast [37].

### 3.3. Diagnosis

Diagnosing syphilis based solely on clinical features is challenging due to the nonspecific symptoms of primary and secondary syphilis, also known as “the great masquerader” for this reason. This difficulty in diagnosis is especially concerning during pregnancy, posing risks for both the mother and, primarily, the fetus [35].

Various laboratory methods, such as darkfield microscopy, immunofluorescent antibody test staining, immunochemistry staining, silver stain, and nuclei acid amplification testing, can be used to evaluate lesions of early syphilis by directly detecting *T. pallidum*. However, these tests are not widely available, and direct fluorescent antibody testing is no longer available in the United States, while nucleic acid amplification tests (NAATs) are available and the results can be used for clinical diagnosis, but no assay has been approved by the Food and Drug Administration (FDA) for commercial use in the United States [30,38].

Therefore, serologic testing forms the basis of diagnosis, similar to nonpregnant individuals [38]. Serological tests for syphilis include treponemal and non-treponemal assays. Treponemal tests detect antibodies against *T. pallidum* antigens, while non-treponemal assays detect antibodies against cardiolipin and lecithin, released during host cell damage.

Various treponemal tests, such as *T. pallidum* hemagglutination assay (TPHA), *T. pallidum* particle agglutination (TPPA), fluorescent treponemal antibody absorption test (FTA-ABS), *T. pallidum* enzyme immunoassay (TP-EIA), and *T. pallidum* chemiluminescence assay (TP-CIA) are available and remain positive even after treatment in most individuals (75–85%) [8].

Non-treponemal tests, such as the Venereal Disease Research Laboratory (VDRL) and Rapid Plasma Reagin (RPR), detect antibodies against biomarkers released from host cells due to *T. pallidum*-induced cellular damage. These tests are used to monitor disease activity and response to therapy. Adequate response to treatment results in a fourfold decrease of RPR titer within a year. In most individuals, RPR will ultimately become nonreactive several months after effective treatment, although sometimes it may remain reactive with low titers (“serofast”) for years. Seropositive pregnant women are infected unless they have documentation of adequate treatment with appropriate serologic response to treatment and RPR titers are low (RPR < 1:4) and stable. Up-trending or persistently high antibody titers may indicate reinfection [39]. Non-treponemal tests are complicated by high false positive rates especially in persons with connective tissue disorders, advanced age, lymphoma, and in infections such as Epstein–Barr virus, hepatitis, HIV, tuberculosis, malaria, and measles [39].

Universal screening for syphilis during pregnancy is recommended by the latest WHO guidelines [31] to enable early diagnosis and subsequent treatment, thereby preventing adverse birth outcomes [36]. The majority of available regional and country-specific recommendations are consistent with this indication [40]. A thorough review of the maternal medical, obstetrical, and social history is crucial in early recognition of risk factors of perinatal syphilis infection, and serological evaluation is crucial [7].

**Figure 1:**
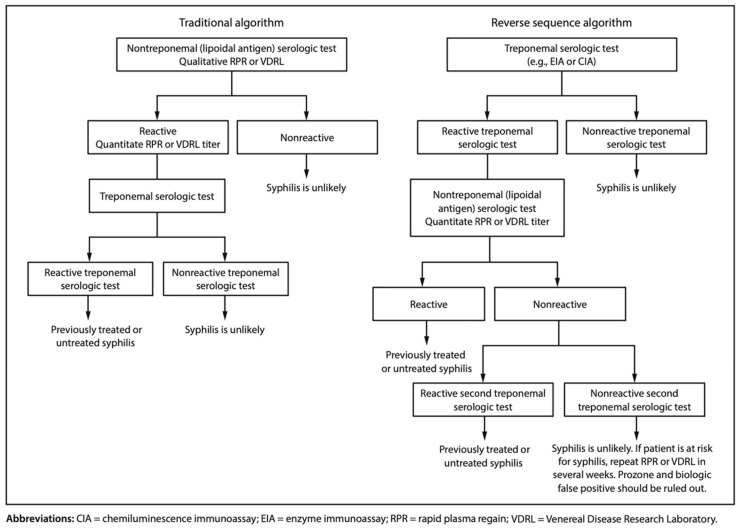
Syphilis serologic screening algorithms [3,9].

Screening involves a combination of treponemal and non-treponemal testing, with the so-called “reverse” sequence algorithm (Figure 1) being frequently recommended as it diagnoses all women with syphilis, including those with latent infections. In fact, in order to prevent CS, even a non-recent infection has to be diagnosed and subsequently treated [31].

The traditional algorithm begins with a non-treponemal immunoassay, followed by a treponemal one for confirmation. In the reverse sequence algorithm, an automated treponemal immunoassay is used first, followed by a quantitative nontreponemal test for confirmation. A patient with reactive results on both treponemal and nontreponemal tests should be considered to have an active infection unless prior treatment resulting in a fourfold decrease from the pretreatment nontreponemal titer is appropriately documented and there is no concern about reinfection according to the timing described in the CDC STI guidelines [30].

Given the time-sensitive nature of effective syphilis treatment during pregnancy, monitoring disease activity and the response to therapy through quantitative non-treponemal tests is essential [30]. Close follow-up is necessary, with repeat testing at specified times, particularly for pregnant women. In fact, non-treponemal titers are assessed at 8 weeks after treatment (and not 6 months after) and at delivery to ensure treatment effectiveness and to prevent reinfection [30]. A fourfold titer increase after treatment may represent treatment failure or reinfection, and a retreatment of the patient with another course of therapy is recommended [41,42]. A fourfold decrease in maternal nontreponemal titers may indicate a maternal treatment response but does not confirm the absence of neonatal infection. When treatment is started in the third trimester, there may not be time for nontreponemal titers to fall by a factor of four before delivery, complicating confirmation of a maternal cure and requiring continued surveillance for the exposed dyad [43].

Timely testing is critical to reduce disease progression and fetus transmission because treatment of maternal perinatal syphilis in the early stages is 98% effective in preventing CS in the newborn [44].

Serologic testing at the first prenatal visit of all pregnant women is recommended by health authorities worldwide [40]. As it can take 10–45 days to be detectable by blood tests, an initial negative test does not guarantee the absence of infection. Moreover, the infection can be acquired later on during pregnancy. For these reasons, pregnant women who are negative in the first test should be screened again later in the pregnancy or at delivery. Historically, it was recommended to repeat the serological evaluation again at 28 weeks of gestation and at delivery if women live in communities with high rates of syphilis or if they have been at higher risk for syphilis acquisition during pregnancy [45,46]. However, recent evidence demonstrated that nearly half of patients did not report any risk factors [47] and repeating the screening for syphilis is superior to a single screening during the first trimester in terms of cost effectiveness and results in an improvement in maternal and neonatal outcomes [48]. Some countries, like Italy, guarantee syphilis screening in the third trimester in all pregnant women regardless of risk factors, which is a better alternative to a risk-based approach [49]. Even in the case of fetal death after 20 weeks of gestation, a syphilis screening should be performed [46]. In conclusion, screening based only on behavioral risk factors may miss a substantial proportion of maternal infection and increase risk for their child.

Screening of pregnant women for syphilis is both cost-effective and cost-saving, even when the prevalence is considerably less than 1% [25]. In fact, the relatively low cost of syphilis screening programs, at USD 0.93–1.44 per person screened, means that such programs are affordable in all but the poorest countries [26]. Screening is extremely cost-effective and affordable, particularly with the new, simple, rapid diagnostic technology. The new tests can also potentially overcome many of the current barriers to services. Finally, synergy between a program for antenatal syphilis testing and a similar program for HIV and malaria testing would consolidate resources and be more cost-effective than any one program alone. The issue for the developing world is, therefore, not whether to screen but how to screen most efficiently [26].

The new, rapid diagnostic tests for syphilis can be performed immediately on-site, allowing infected women to be identified and treated at a single visit, significantly increasing the numbers of women treated [31].

In the case of a positive treponemal test and a quantitative non-treponemal test, the woman must undergo a thorough examination for staging of the infection and ultrasonography to identify signs of congenital infection in the fetus, even if no individual sonographic sign or pattern of signs is pathognomonic for fetal syphilis [50]. On the other side, in fetuses with ultrasound abnormalities suggestive of congenital infection, syphilis must be considered as part of the workup [50]. In fact, ultrasonographic evidence of intrauterine infection can be detected after 18 weeks of gestation, when the fetus is able to mount an immune response to *T. pallidum* infection, and includes fetal hepatomegaly (in 80% of cases), anemia as suggested by the peak systolic velocity of the middle cerebral artery (33%), placentomegaly (27%), polyhydramnios (12%), and nonimmune hydrops (10%) which led to preterm delivery in 80% of cases [51]. Unfortunately, the absence of ultrasonographic abnormalities does not rule out congenital infection that can be diagnosed at birth in 12 to 15% of at-risk fetuses with a negative ultrasound evaluation [52]. Fetal anemia and related hydrops generally resolve within 3 weeks after maternal treatment, with subsequent normalization of fetal liver and placental measurements occurring up to 15 weeks after treatment [52]. Fetal ultrasonographic abnormalities should be monitored serially for better pediatric management at delivery.

Given the limited sensitivity of dark-field microscopy (when available) and nucleic acid amplification testing on amniotic fluid, there is no indication in the available guidelines for the performance of amniocentesis as an implication for a different therapeutic approach [52].

Contact tracing and treatment of partners are also vital components of preventing reinfection in the pregnant women [31].

### 3.4. Treatment

Syphilis in adults is easily cured. Penicillin is the first-line therapy for all stages, with limited evidence available for alternative antibiotics [30]. The choice of penicillin form (benzathine or aqueous crystalline), dose, and duration depends on the stage and site of infection [30].

The only antimicrobial agent for treating syphilis during pregnancy proven to be both safe and efficacious is penicillin, administered according to the clinical stage of the infection with the same regimens recommended in nonpregnant adults [31]. It should be widely available in primary healthcare settings [53].

According to the WHO [31], treatment consists of a single dose of long-acting benzathine penicillin G (BPG, 2.4 million units intramuscularly) for primary, secondary, and early latent syphilis, without the benefits of an additional dose, or three weekly doses (7.2 million units total) for late latent or tertiary syphilis. The recommended treatment for neurosyphilis is aqueous crystalline penicillin G (3–4 million units every 4 h intravenously for 10–14 days) because BPG does not result in treponemicidal levels of the antibiotic in the cerebrospinal fluid (CSF) [31]. If the interval between doses exceeds 9 days, the treatment is considered to be inadequate and should be reinitiated. Moreover, it has to be completed by 4 weeks before delivery to be effective [30,31,43].

Treatment may be complicated by the Jarisch–Herxheimer reaction in up to 40% of pregnant women—a complex allergic response to antigens released from dead micro-organisms, characterized by cramping, pyrexia, and myalgias. This reaction occurs 1 to 2 h after penicillin administration and resolves spontaneously in 24–48 h. As it may cause fetal distress and uterine contractions, continuous fetal heart rate monitoring for 12 to 24 h after treatment should be considered to confirm fetal well-being.

No treatment options exist for pregnant women who are allergic to penicillin as doxycycline is contraindicated during pregnancy. Therefore, in the case of a history of penicillin allergy, evaluation with skin testing or an oral penicillin challenge is encouraged in pregnant women [54], followed by treatment with penicillin with the same regimen described below due to the lack of alternate therapy [55]. However, this recommendation is present only in a few countries’ guidelines [40,56]. A history of severe hypersensitivity syndrome, such as Stevens–Johnson syndrome, warrants consultation with an allergist [30].

Shortages of BPG have been registered worldwide [57]. In this situation, a common reported error is the administration of aqueous crystalline penicillin intramuscularly, which is a short-acting form of penicillin [58].

If alternative regimens are used during pregnancy, both for penicillin allergy or penicillin shortage, the newborn should be evaluated and potentially treated for CS [30].

Unfortunately, international guidelines still present considerable discrepancies in terms of the optimal therapeutic regimen; in fact, alternative regimens to BPG were listed in 42 (68%) guidelines, primarily from Africa and Asia, and only 20 specified that non-penicillin regimens are not proven effective in treating the fetus [40].

When provided early in pregnancy, antenatal treatment is highly effective at reducing the risk of congenital syphilis by 97%, stillbirths by 82%, preterm delivery by 64%, and neonatal death by 80% [28]. Even in women with syphilis of long duration who themselves would benefit from three weekly doses of penicillin, a single dose of penicillin prevents infection in the fetus. Birth outcomes for such women are similar to those for women without syphilis [59].

In the case of no syphilis screening and treatment, the MTFT occurs transplacentally; therefore, the newborn is already affected at the time of birth. For this reason, there is no indication for cesarean section unless the presence of active lesions along the birth canal were documented during the physical examination of the pregnant woman at the time of delivery [60].

## 4. Management at Birth

The diagnosis and treatment of CS in infants is considerably more difficult than the diagnosis and treatment of infected pregnant women.

### 4.1. Clinical Features

CS is a serious but preventable disease which can lead to fetal loss, stillbirth, neonatal death, and congenital infection. Based on the age when clinical symptoms appear, CS can be classified as early when the infection presents within the first 2 years after birth (but most commonly within 3 months after delivery) or late when the onset is later in life [5]. Large, thick, and pale placenta and abscess-like foci of necrosis in Wharton’s jelly centered around the umbilical vessels (necrotizing funisitis with “barber-pole appearance”) can be observed in CS. Early CS can be asymptomatic in approximately 70% of cases (that is the condition when newborns and infants seem to be clinically normal, with a normal physical examination and no signs of central nervous system involvement, but have positive serological tests) or lead to manifestations during pregnancy (as described above) [50]. In the neonatal period, usually the primary lesion is lacking in contrast with what happens in adulthood, and CS begins as a secondary form, as it is septicemia with a possible fulminant sepsis picture [5]. The other most common manifestations are described in Table 2 [5].

Liver transaminases may worsen after initiation of penicillin therapy and may represent a localized Jaresch–Herxheimer reaction [5]. The pseudo paralysis of Parrot (decreased range of movement due to painful periostitis) may materialize [5,50]. Lesions in the long bones are pathognomonic of CS with moth-eaten appearance because of demineralization [61]. Hypoxic ischemic encephalopathy, persistent pulmonary hypertension of the newborn, and disseminated intravascular coagulation have also been reported [62]. The Hutchinson’s triad, which is specific for congenital syphilis, includes Hutchinson’s teeth, interstitial keratitis, and cranial nerve VIII nerve deafness. 

Clinical manifestations of CS, as described above, especially the early ones, are not specific. Thus, maternal serology is essential to diagnose the infection in the children and to treat it.

### 4.2. Diagnosis and Management

CS is defined as the transmission of a maternal syphilis infection through the placenta to the fetus either in the womb or during childbirth [26].

All infants born to mothers with a known perinatal syphilis infection or positive syphilis screening (combination treponemal and nontreponemal) during pregnancy or at delivery should undergo screening for congenital syphilis prior to discharge from the birth hospital (and all mothers with an unknown evaluation should undergo screening for syphilis prior to discharge from the birth hospital [26]).

The diagnosis and subsequent management of infants and children is based on maternal information (history of syphilis infection and treatment, RPR results during pregnancy and at the time of delivery, and risk factors for infection/reinfection), infant physical exam findings, and infant workup. However, the diagnosis of CS can be challenging, with no single diagnostic test available and multiple limitations in the current testing modalities, including maternal antibody transfer obscuring the diagnosis [39]. The commonly used testing modalities include paired maternal and newborn serology, PCR, and placenta histopathology [39]. In addition, newborn clinical features are evaluated.

Since anti-treponemal IgM does not cross the placenta, if detected in neonatal serum, it is suggestive of congenital infection, but a nonreactive result does not exclude the diagnosis [63].

Since maternal treponemal antibodies can be passively transferred to the fetus in the absence of active neonatal infection, evaluation of this marker is not helpful [39]. Nontreponemal IgA and Igg antibodies are more useful for neonatal diagnosis, though their sensitivity is reduced because they passively cross through the placenta [39]. The diagnostic criteria include neonatal nontreponemal titers four times higher than maternal ones, even though the absence of this difference does not rule out a diagnosis of CS [39].

Newborns meeting the criteria for confirmed or highly probable syphilis should undergo evaluation for neurosyphilis. Cerebrospinal fluid (CSF) test results are difficult to interpret, since nontreponemal antibodies may be passively transferred from plasma unto the CSF. The sensitivity of a CSF VDRL test is approximately 50%, with 90% specificity [39,64].

Classification of risk of CS is described in Table 1.

Histopathologic examination of the placenta is a valuable adjunct to the contemporary diagnostic criteria used to diagnose CS, with a sensitivity of 67 to 82% and specificity of 58% [65].

PCR evaluation in newborns is not routinely used for the diagnosis of CS because it lacks evidence of an ideal sample and evaluation is frequently based on small samples [39].

In late CS, diagnosis is based on an abnormal physical examination consistent with CS and positivity of treponemal and nontreponemal tests. The evaluation consists of the following: complete blood count with differential and platelet count, any other clinically indicated test (liver function test, auditory brain stem response, ophthalmologic exam), and CSF analysis for VDRL, cell count, and protein.

### 4.3. Treatment

Due to the scarcity of diagnostic tests for newborns and their aforementioned limitations, coupled with the critical importance of not missing a diagnosis, treatment decisions typically rely on a combination of factors, such as the mother’s syphilis status, the adequacy of maternal treatment, the time between maternal treatment initiation and delivery (with less than 30 days considered insufficient), comparison of maternal and neonatal serologic titers at delivery, and the presence or absence of clinical, laboratory, and radiographic evidence of syphilis in the newborn [30].

Infants born to seroreactive women who have not received appropriate treatment should be treated according to specific protocols [30,66]. Newborns meeting the criteria for confirmed or highly probable syphilis should receive weight-based intravenous aqueous crystalline penicillin G treatment (Table 3), even if ampicillin and gentamicin have been previously administered for suspected neonatal sepsis [30]. This regimen, which consists of 50,000 units/kg administered every 12 h for the first 7 days of life and every 8 h thereafter for a total of 10 days, stems from extensive clinical experience and two randomized clinical trials conducted in 1989 and 1997. An alternative regimen is represented by intramuscular administration of procaine penicillin G at a rate of 50,000 units/kg in a single daily dose for 10 days [30]. The intramuscular route is very painful and the drug is lacking globally, so this regimen has to be used only in limited cases.

The administration of this prolonged and intensive intravenous penicillin regimen for treating neonatal syphilis may pose challenges for patients and families and may result in high bed occupancy or reduced adherence in certain settings. Hence, prevention of CS through universal screening of pregnant women early in pregnancy and subsequent treatment, if warranted, is far preferable.

In cases of late CS diagnosis, children should be treated with weight-based intravenous aqueous crystalline penicillin G, given as 50,000 units/kg every 4–6 h for 10 days.

## 5. Follow-Up

Follow-up monitoring is essential. All children with reactive nontreponemal tests at birth should be monitored serologically every 2 to 3 months to ensure normalization of tests results. Passively transferred maternal nontreponemal antibodies may persist until 15 months of age. However, in most uninfected infants, nontreponemal titers typically normalize by 6 months of age [39]. If elevated titers persist beyond 6 months, further evaluation and treatment may be necessary [30]. High rates of failure to follow-up, reaching up to 65% in certain U.S. regions, increase the risk of missed or delayed diagnoses and subsequent neonatal complications [67]. Lower rates of failure to follow-up have been reported in other settings, possibly due to differences in healthcare organization, such as in Italy, where a recent study reported a 12.5% failure rate [68]. The need for a specific and prolonged follow-up is indisputable because [64] children with CS are at risk of late and persistent intellectual disability, hearing impairment, and skeletal abnormalities [69].

## 6. Further Directions

### 6.1. Eliminating Missed Opportunities for Prevention

Current epidemiological data show a re-emergence of syphilis worldwide, in vulnerable groups and even in the general population. This could be related to an underestimation of this infection associated with nonspecific clinical manifestations and with the decrease in the use of condoms eventually associated with the use of PrEP (Pre-Exposure Prophylaxis) as an HIV prevention method. Moreover, often there is the stigmata that this infection is more frequent in specific groups of the population and in low- and middle-income countries, so that the general population especially in high-income countries consider themselves not at risk.

The elimination of perinatal syphilis is feasible through timely diagnosis and treatment during pregnancy [26]. Prevention of congenital syphilis requires robust antenatal healthcare systems with rigorous surveillance, screening, testing, and treatment. Strengthening national surveillance and information systems is crucial for accurately monitoring cases of syphilis and congenital syphilis.

Common missed opportunities for preventing CS include delayed or absent prenatal care (found in 42% of perinatal syphilis cases), inadequate maternal treatment (31%), late identification of maternal seroconversion during pregnancy (14%), and prenatal care without syphilis testing (8%) [70]. In the U.S., 88% of the 3.761 cases of CS reported in 2022 resulted from a lack of timely testing, inadequate treatment, or both in pregnant women. Populations with the highest syphilis prevalence tend to have the lowest antenatal care coverage [16]. Missed opportunities vary by U.S. regions, underscoring the need to tailor prevention strategies to local contexts. Given the limited utility of individual risk behaviors in predicting syphilis, approaches should be refined to identify specific patients and contextual characteristics that make third-trimester syphilis screening or screening at delivery cost-effective.

Clinical suspicion of syphilis infection at any pediatric age warrants a comprehensive evaluation by a pediatric infectious disease specialist. Programs supporting maternal and neonatal adherence to longitudinal follow-up are crucial for reducing missed or delayed diagnoses and subsequent morbidity.

With the current neonatal risk scenario described by the CDC [30], both overtreatment of uninfected newborns and late diagnosis in infected ones are possible. To better identify infected newborns early, new diagnostic approaches which are capable of detecting *T. pallidum* infection at birth are needed. Several *T. pallidum* surface or subsurface lipoprotein targets have been evaluated in adults for use in diagnostic research testing serum, as well as noninvasive specimens, such as pharyngeal and rectal mucocutaneous swab specimens [71,72]. Near-future use in clinical practice is desirable. Early diagnosis at birth would substantially reduce the burden of follow-up, which disproportionately affects patients with socioeconomic barriers [73]. Financial stability plays a major role in the access to prenatal medical care for many disadvantaged persons. Unstable housing, language barriers, lack of insurance, and transportation are well-reported barriers to prenatal care [74]. However, syphilis is not only spread in high-risk groups of the population [68]. For this reason, universal re-screening during pregnancy could be a better alternative to a risk-based approach.

The inclusion of point-of-care testing in surveillance definitions may facilitate better comparisons between countries, though challenges exist due to varying access to diagnostic testing. Key recommendations should include the frequency of syphilis testing, partner testing, and identification of region-specific high-risk pregnancies [31].

The use of unique case definitions would facilitate better comparisons between countries, but it is challenging due to varying access to diagnostic testing between countries.

The treatment of male partners is an important contributor to breaking the chain of maternal infection and reinfection that contributes to CS. However, despite increased efforts to improve syphilis testing and treatment, strategies to improve the treatment of sexual partners are failing [75]. Encouraging men to accompany women to the antenatal clinic and testing both may address the urgent need to treat partners. However, they expressed an unwillingness to receive syphilis treatment because of fear of injections and loss of dignity [75].

### 6.2. Treatment

Regarding therapy in mothers and newborns, several aspects need improvement in the near future, particularly the development of alternative drugs, as summarized in Table 4.

Effective non-penicillin-based regimens are necessary for pregnant women with true penicillin allergy and would provide alternative treatments during penicillin shortages. Non-parenteral drugs might also be more conducive to administration and outpatient management.

In case of benzathine penicillin G shortage, as is currently happening globally [57], prioritizing its administration for pregnant women and newborns is advised [30]. Widespread point-of-care testing is estimated to increase the number of doses required for pregnant women, from 414,459 in 2019 to 1,078,428 in 2030 [83].

The limited number of producers for penicillin’s active ingredients, along with a rise in demand, increases the likelihood of future shortages. There are zero other options when there is no BPG to treat pregnant women and patients with neurosyphilis. Consequently, in the face of BPG shortages, several agencies have advised to treat uncomplicated non-pregnant patients with doxycycline to preserve the supply of BPG and maintain stock for high-priority patients [84].

Another issue related to BPG is the reporting of a few cases of penicillin treatment failure [85,86,87], especially in people living with HIV, but differentiating between reinfection and relapse remains challenging. While penicillin-resistant T. pallidum has not been documented, the historical inability to culture the organism limits drug susceptibility testing. The emergence of resistance to penicillin in *T. pallidum* is considered very unlikely as it would require complex mutational processes that do not seem to have occurred after over 70 years of continuous use of this antibiotic. However, there is a possibility of penicillin-resistant strains, as genomic analysis indicates potential targets like penicillin-binding proteins (PBPs) that could undergo mutations reducing their affinity for penicillin [88].

Penicillin G procaine, a prior treatment option for neonates with possible, confirmed, or highly probable CS, is no longer available, because it has been discontinued by the manufacturer [30].

Research is needed to expand therapeutic options and develop future treatment strategies for challenging stages and situations in syphilis management. Notably, randomized controlled trials evaluating treatment regimens in pregnant women and neonates are currently lacking.

A major obstacle to testing alternative antibiotics for syphilis was the long-standing inability to culture the causative agent *T. pallidum*. However, a system for culturing it was established in 2018, allowing for the determination of MICs of a broad repertoire of antimicrobial agents. In vitro studies have shown anti-treponemal activity for the following antibiotics: penicillin G (MIC ≤ 0.003 mg/L), doxycycline (MIC 0.10 mg/L), ceftriaxone (MIC 0.0025 mg/L), several oral cephalosporins (cefixime, cefetamet, cefuroxime; MIC 0.01–0.03 mg/L), amoxicillin (MIC 0.02 mg/L), linezolid (MIC 0.5 mg/L), dalbavancin (MIC 0.125 mg/L), and spectinomycin (MIC 0.1 mg/L) [81,82,89].

Azithromycin (2 g in a single oral dose) was previously recommended for early syphilis after showing similar effectiveness to BPG [56]. However, its use is no longer recommended due to an increasing level of resistance. In fact, macrolide use for unrelated infections (e.g., oral, skin, respiratory, and genital infections) shortly before the *T. pallidum* infection creates selective pressure leading to the selection of resistant strains. Global reports indicate a high prevalence of macrolide resistance (ranging from 64% to 100%) with pregnant women receiving azithromycin resulting in congenital syphilis and neonatal deaths [90,91].

Another possible oral therapy consists of a 14-day course of oral amoxicillin with and without probenecid which has shown an overall treatment efficacy of 94–95%, including in early and late syphilis and in people with HIV. However, in a case series of pregnant women treated with oral amoxicillin alone, the benefit was limited to those with early syphilis. In fact, there were no cases of CS in infants born to women with early syphilis, but this was diagnosed in 33% of infants born to women with late syphilis [76,77,78]. For these reasons, oral amoxicillin is currently not recommended for the treatment of early syphilis and should be specifically avoided during pregnancy.

A possible alternative was the intravenous administration of ceftriaxone for 10 days, although its use in pregnancy has been limited to case studies and non-randomized studies and no strong recommendation on its use in preventing congenital syphilis is currently possible [56]. Ceftriaxone is given only once daily and would therefore require fewer doses than an intravenous penicillin-based regimen, which would allow for ambulatory treatment. However, ceftriaxone is given as a 10-day intravenous or intramuscular course, which presents a substantial burden to healthcare systems and might not be suited to LMICs. Particularly in this setting, oral regimens could be useful. However, no evidence exists to support the use of oral agents in pregnancy or neonates.

Oral doxycycline is recommended as an alternative therapy for the treatment of syphilis in nonpregnant adults [92]. Although short-term administration (3 weeks) of doxycycline during breastfeeding is not contraindicated, adherence may be compromised because of hesitancy related to the potential adverse effects among breastfeeding infants, such as enamel staining and skeletal defects.

Linezolid shows promise for the treatment of syphilis based on in vitro studies indicating anti-treponemal activity [81]. However, due to the rapid spread of macrolide resistance in *T. pallidum* worldwide and the potential impact of single point mutations on the same target gene as azithromycin, caution is advised against the widespread use of linezolid. An ongoing randomized trial is testing its efficacy for primary and secondary syphilis [80].

Finally, zoliflodacin is an effective antibiotic against N. gonorrhoeae, and it may also have efficacy for syphilis with an MIC of 2 mg/dL [82]. The anti-treponemal activity may be explained by the conservation of key amino acid residues in the T. pallidum DNA gyrase sub-unit B protein, similar to that of the N. gonorrhoeae GyrB protein [93].

Key additional questions pertain to the minimal effective duration of third-generation cephalosporin, the effectiveness of single-dose penicillin in some high-risk neonates, and the treatment duration of asymptomatic versus symptomatic infection.

In conclusion, the absence of proven alternatives to penicillin for treating syphilis during pregnancy underscores the need to ensure access to penicillin supplies to prevent cases of CS due to inadequate treatment availability [57].

No vaccines are currently in human clinical trials, and more research is needed to establish the optimal vaccine platform or target antigen. Knowledge about immune correlates of protection from syphilis infection is poor. Current evidence suggests low genetic diversity in most syphilis genes, although there is variation within outer membrane proteins, which appears to be a major immunogenic surface molecule. More studies are needed to evaluate genetic diversity in high-prevalence areas so that antigenic variations can be considered in the vaccine design [94].

## 7. Conclusions

### Congenital Syphilis Is a Largely Preventable Disease

Diagnosis in pregnant women is often missed because of lacking screening programs or non-adherence to them when existing. Implementing screening programs is the key for the prevention of CS and needs specific funding and widespread testing among all pregnant women, not only in high-risk ones. Moreover, the serological evaluation has to be performed in the first trimester of pregnancy but also repeated in the third one or at delivery. Implementation of a rapid but accurate diagnostic test is fundamental to identify all the infected pregnant women. No women should be discharged after delivery without a serologic evaluation for syphilis.

Moreover, every effort should be taken in identifying infected newborns in order to reduce the administration of parenteral antibiotic therapy, with subsequent prolonged hospitalization, in highly probable or possible cases.

Finally, future research is needed for identifying an alternative to benzathine penicillin G that is suitable for pregnant women, preferably administrable orally, considering its global shortage.

Efforts should be made worldwide to implement appropriate management during pregnancy and achieve the WHO’s target rate of under 50 cases per 100,000 live births.

## Figures and Tables

**Table 1:** Risk factors for perinatal syphilis infection [22].

Type of Risk Factor	Description
At individual level	High risk sexual behaviors; Substance abuse; Belonging to vulnerable groups either due to geographic location or race/ethnicity; Poor health literacy; Language barriers; Inadequate prioritization of personal healthcare and poor utilization of available resources; Stigma and fear of judgment; Lack of health insurance
Community level	Inadequate access to healthcare; Limited number of clinicians accepting Medicaid; Inadequate medical knowledge on syphilis among clinicians and limited guidance; Judgmental and stigmatic approach; Poor provision of sexual health education
System level	Poverty; Structural and systematic racism; Homelessness and housing insecurity; Inadequate focus and resource allocation to rural and remote areas; Lack of funding; Inadequate public health infrastructure; Integration of health information systems to provide information to health policymakers

**Table 2:** Clinical, laboratory, and radiographic findings in congenital syphilis.

Early congenital syphilis (<2 years of age)	
*Clinical and physical examination findings*	normal; stillborn; preterm; nonimmune hydrops fetalis; intrauterine growth restriction; small for gestational age; fever; hepatomegaly * with or without jaundice; splenomegaly *; rash *; rhinitis (snuffles); mucous patch; condylomata lata; adenopathy (epitrochlear nodes); pseudoparalysis of Parrot; chorioretinitis; cataract; irritability; cranial nerve palsies; seizures; pancreatitis; myocarditis; gastrointestinal malabsorption
*Laboratory findings*	anemia, thrombocytopenia *; hypoglycemia; liver transaminitisc and direct hyperbilirubinemia; cerebrospinal fluid pleocytosis, elevated protein content, reactive VDRL/RPR test; proteinuria (nephrotic syndrome); hypopituitarism (diabetes insipidus)
*Radiographic findings*	periostitis; osteochondritis *; pneumonia alba
**Late congenital syphilis (>2 years of age)**	
	Hutchinson’s teeth; Mulberry molars; interstitial keratitis; optic nerve atrophy; healed chorioretinitis; rhagades; gummas; cranial nerve VIII nerve deafness
	intellectual disability; hydrocephalus; seizures; juvenile general paresis; cranial nerve palsies
	frontal bossing; saddle nose deformity; protuberant mandible; short maxilla; high palatal arch; perforation of the hard palate; saber shin; sternoclavicular joint thickening (Higouménakis sign); Clutton’s joints (painless swelling of the knee)

* Prominent features.

**Table 3:** Adapted evaluation and management guidelines based on CDC and AAP risk-stratification algorithms [1,3,13].

Scenario (CDC) Risk Category (AAP)	Clinical History and Examination	Evaluation	Treatment
Scenario 1, Category: Confirmed proven or highly probable congenital syphilis	Abnormal physical exam OR RPR titer ≥4-fold maternal titer	CSF analysis (cell count, protein, VDRL), CBC, Long bone radiographs, Other *	Intravenous Penicillin G x10 days (regardless of evaluation results)
Scenario 2, Category: Possible congenital syphilis	Normal physical exam AND RPR titer <4-fold maternal titer AND Maternal treatment none/unknown/inadequate or initiate <30 days before delivery	CSF analysis cell count, protein, VDRL, CBC, Long bone radiographs	Intravenous Penicillin G x10 days (if evaluation is abnormal, uninterpretablem incomplete, or follow-up uncertain) OR Intramuscular Benzathine Penicillin x 1 (if evaluation and follow-up are certain)
Scenario 3, Category: less likely congenital syphilis	Normal physical exam AND RPR titer <4-fold maternal titer AND Maternal treatment adequate and initiated ≥30 days before delivery and no concern for reinfection	None	Intramuscular Benzathine Penicillin x 1 (if follow-up uncertain) OR No treatment with follow-up titers (follow-up certain)
Scenario 4, Category: unlikely congenital syophilis	Normal physical exam AND RPR titer <4-fold maternal titer AND Maternal treatment adequate before pregnancy	None	No treatment with follow-up RPR titers (if infant RPR positive) OR Intramuscular Benzathine Penicillin x 1 (if follow-up uncertain)

CSF: cerebral spinal fluid; CBC: complete blood count; RPR: rapid plasma reagin; VDRL: Venereal Disease Research Laboratory Test; * other tests as clinically indicated (e.g., liver function tests, neuroimaging, ophthalmologic exam, and auditory brain stem response).

**Table 4:** Strategies to mitigate resistance development and spread (modified by Mitjà O et al. [56]).

Improved Use of Current Drugs	Drug Discovery
- Development of new therapeutic options for patients with penicillin allergy - Development of new treatments with good distribution to the central nervous system for neurosyphilis and in utero for congenital syphilis	- Amoxicillin (mixed results, further evidence required) [76,77,78] - Cefixime (potential treatment for early syphilis; ongoing phase II RCT) [79] - Linezolid (potential oral treatment for neurosyphilis; concern on resistance; ongoing RCT for primary and secondary syphilis) [80,81] - Zoliflodacin (in vitro evidence) [82]
